# Ninth International Medical Congress, Washington, D. C., September, 1887

**Published:** 1888-02

**Authors:** 


NINTH INTERNATIONAL MEDICAL CONGRESS, WASHINGTON D. C. SEPTEMBER, 1887.

SECTION XVIII, DENTAL AND ORAL SURGERY.

REPORTED FOR THE INDEPENDENT PRACTITIONER, BY "MRS. M. W. J."

CONTINUED FROM PAGE 29.

THURSDAY AFTERNOON SESSION.

Dr. W. W. Allport, of Chicago, in the chair.

Dr. E. S. Talbot, of Chicago, read a paper on the "Etiology of Irregularities of the Jaws and Teeth."

Dr. Talbot thought that comparison of the teeth and jaws of to-day with those of earlier dates showed that irregularities were more common at present, and were also on the increase, the causes for this meriting greater attention and observation. He considered the main source of irregularities to lie in the unequal development of the maxillary bones, which grow independently of each other, the different forms of irregularity met with being due to the arrested development of one or more of the separate bones forming the arches. His paper was largely devoted to statistics founded on observations made in various institutions for idiotic and feeble-minded children, arrested development of the maxillary bones with vaulted or V-shaped or saddle-shaped arches being characteristic of these unfortunate classes. As far as possible a study was also made of the hereditary or other causes of these malformations.

At the conclusion of this paper the regular order of business was suspended in honor of the presence of Dr. N. S. Davis, President of the Congress, who was introduced to the Section by President Taft, as the one individual above all others to whom the dental profession was indebted for the position it occupied in the International Medical Congress.

Dr. Davis addressed the Section. He said the day had gone by for founding sects and schools on theoretical dogmas; that medicine, to be worthy the name of a science, must be founded on the scientific investigation of accumulated facts, and conclusions drawn by inductive logic; that the dental branch did not differ from the other departments, all aiming at the same goal, and, through harmonious action, attaining a scientific plane. He said that as long ago as 1865 he had said that the teeth and the jaws were as important as the eye, the ear, or any other part of the human organism, and a knowledge of how to treat these diseases required the same scientific knowledge of the principles of medicine that any other branch demanded. He hoped to live to see the last vestige of the so-called "schools of medicine" abolished; when there should be no homœopaths, no eclectics, no allopaths, but all stand on a broad platform as doctors of medicine, without any pathy, as co-workers in the great field of science.

Prof. Busch, of the Dental Department of the University of Berlin, who had arrived during the address of Dr. Davis, was introduced, and expressed his gratification at what he had just heard; he said that he was laboring to secure the same recognition in Germany, and not without hope of success.

Dr. E. H. Angle, of Minneapolis, read a paper entitled "Notes on Orthodontia, with a New System of Regulation and Retention." He said that in the work of regulation but five movements could be made—forward or backward, and inward or outward in the line of the arch, and partial rotation, these being all governed by the same principles, and easily accomplished by means of jack-screws for pushing, traction-screws for pulling, and a simple rotating appliance of piano wire bent at one end into the form of a hook. The teeth to be moved by Dr. Angle's method have a band cemented around each one, and also on the teeth of resistance, a piece of joint wire one-fourth of an inch in length being soldered to each band. This he calls "banding and piping" the teeth. A piece of gold-plated wire is threaded through all the pipes around the arch, against which the base of the jack-screws, etc., is applied, modified in different ways to suit the case in hand. The system seemed very simple and yet most effectual. The methods of applying the force were, in many instances, exceedingly ingenious, and yet uncomplicated, and the reading of the paper was heartily applauded.

In the discussion of this paper Drs. Farrar, New York; Morrison, St. Louis; Brown, Flushing, L. I.; Talbot, Chicago; and others, denied that the system of Dr. Angle was either new or original. Drs. Haskell, of Chicago, and Bailey, of Minneapolis, thought that he had made new and original applications of old principles, and in so far his method was new.

Dr. W. C. Barrett objected to the discussion, as foreign to the work of the Section, which should be restricted to the consideration of principles and not appliances. He said that this Congress was not a court before which to try and settle questions of priority of invention. He was seconded by Dr. Allport and upheld by the President.

Dr. Barrett said that one force which, to his apprehension, was potent in the production of peculiarities and irregularities, and not alluded to by Dr. Talbot in his paper, was simply mal-occlusion of the teeth. Many cases of the saddle-shaped jaw can be traced to this cause. Heredity was also a very potent factor. Dr. Barrett did not agree with the statement that the examination of prehistoric skulls showed change of type of the jaws or teeth. On the contrary, he thought there had been but very little change in three thousand years. He himself had tabulated the results of an examination of more than two thousand skulls, the most of them belonging to prehistoric races, and he had found that comparatively little change could be observed. The same diseases were prevalent three thousand years ago that we to-day are combating, and there was about the same relative number of supernumerary and of rudimentary teeth as now. Irregularities were less common, perhaps, because the mingling of types was more infrequent.

FRIDAY MORNING SESSION.

Prof. Busch, Director of the Dental Institute of the Royal University of Berlin, read a paper on "The Comparative Pathology of the Teeth, with Special Reference to the Tusk of the Elephant."

This paper was illustrated by a very large collection of pathological specimens of ivory, which were handed around for examination after Prof. Busch had pointed out and explained the different forms of diseased conditions and the probable or possible causes. In some there were defects resembling caries in human teeth, both with and without pulp connection. In some the pulp had been protected through nature's efforts, by the deposition of lime-salts, similar to the protective or secondary dentine formed in similar cases in human teeth. Some defects of a peculiar character were ascribed to the work of some boring animal, from which devitalization had resulted. Although caries is seldom present in the dentine of the larger animals, in these specimens alveolar abscess was frequent. In some specimens the pus cavity was separated from the pulp chamber by a wall of secondary dentine; others showed the abscess in the pulp itself, with secondary dentine, rendering the chamber very circumscribed and crowded. In several specimens there was no visible sign outside of the cavity existing in the dentine; as it is impossible that an abscess should have formed in the solid dentine, it must have originated in the pulp chamber, the pressure of the pus upon the walls of the chamber creating an opening into the dentine, breaking down the canaliculi, the pathway being subsequently obliterated by the elongation of the tusk, which never ceases during life. Other specimens showed an irregular development of dentine, the lime-salts being deposited in such a manner as to form round balls, like bullets, quite separated from the surrounding normal dentine, giving the appearance of a ball in a socket; probably some process of desiccation had separated the ball from its surroundings. Dental nodules were shown in the pulp chamber, some still attached to the walls; in others the connection had evidently been severed. Another specimen showed the union of a fracture with a large callus, the healing process having been sufficient to re-establish union of the parts. This is also sometimes seen in human teeth. In one specimen an iron bullet had traversed the dentine, pierced the pulp, and lodged in the opposite side of the chamber, the pathway being filled with secondary dentine, though the outer wall had evidently been shivered and fractured. In another very interesting specimen the bullet had not penetrated to the pulp chamber, and yet the point of entrance was closed by secondary dentine. It has generally been supposed that the formation of secondary dentine was exclusively due to pulp function, but in this case it must have had its source in the peridental membrane, as the pulp had not been reached.

Prof. Busch also introduced a little novelty of great advantage in the painless, quick removal of small moles or warts. It consisted of a series of cutting cylinders of different sizes: one of the exact circumference of the mole or wart should be selected, and by a rapid rotation in the dental engine made to cut the outer skin, the remainder being clipped with scissors, any dressing for hemorrhage being applied; at the end of a few days nothing will be visible but a small white scar, scarcely perceptible. The largest size cylinder was 1½ centimetres. Above that size it is not advisable to remove by such an operation. He passed around a bottle of specimens removed during his last course of lectures at the University, Berlin.

Dr. Wm. H. Atkinson opened the discussion of this paper. He said the overwhelming presentment of specimens represented a lifetime of observations in the workshops of ivory-workers among the refuse rejected by them as worthless, and yet so invaluable to the scientific mind as a revelation in the settlement of disputed questions in histology. His greatest regret was that he could not speak German, that he might thank Prof. Busch, in his own language, for this great contribution. He regretted, however, that he could not accept all the deductions drawn from his observations by Prof. Busch. From his own observations in the shops, he thought that old age gave the globular formations, from the debility of the building powers. We know the why of nothing, and only the *how* in a very small degree. In the globular formations, only sufficient lime-salts are deposited to complete the consolidation of a point in the centre. Concentric rings of secondary dentine subsequently formed around this point. Dr. Atkinson said that the statement that in the young African elephant the tusks are tipped with an enamel-cap, smelled as though the studies had been made on the teeth of eels; the enamel-tipped tusk was only a reminiscence of the *anguillula,* before there was an elephant; he, himself, undoubtedly developed deciduous teeth in the cetaceous stage, which were shed before he was born, but he had no recollection of his sensations and observations at that epoch. (At this point Prof. Busch, rather to the confusion of the speaker, handed him a very small tusk which was covered by an enamel cap). In many of the specimens Dr. Atkinson said he failed to see the secondary dentine spoken of. They told a different story to him. The original lime-salts had been melted down, but not carried away; they were formed into a magma, and then again consolidated, but not conforming to the original structure. Many of the so-called pus cavities were not abscesses at all, but divisions of the pulp chamber. Wherever there were perfectly smooth walls there had been pulp tissues.

Dr. Friedrichs said that he would not presume to criticise the able demonstration of Prof. Busch, but there was one point that he did not understand, and of which he wished an explanation from Prof. Busch. The spaces seen in the specimens were said to have been produced by abscesses; one was entirely enclosed; in human teeth we sometimes have a deposit of secondary dentine that partially cuts the pulp off; sometimes when globules are developed, or imperfect formations, we have a cornua recurring high up, the pulp located where we had not expected it. To his mind pus was always associated with an abscess, and the inference he drew was that there must have been a mass of encysted pus. Nomenclature had so much to do with these questions that he would like Prof. Busch's definition of what he meant when he said abscess.

Prof. Busch replied that he had not pretended positively to account for all the abnormal conditions; he had only made suggestions as to the probable interpretations. Some of the cavities might be pulp chambers, and others might be abscess cavities. Where there is irritation and inflammation there may be formation of pus, and then a formation of secondary dentine making a separating wall. His reason for believing that many of the cavities were abscesses was because in cutting them open he encountered the unpleasant, putrid, penetrating odor so well known and promptly recognized.

Dr. Friedrichs said the conditions of growth were so different that the functions of the pulp of the tooth of an elephant were very different from the human tooth. If pus forms in the human, the pulp is destroyed; he could not conceive how it could then continue to perform its functions in the formation of secondary dentine.

Dr. W. C. Barrett said that the tooth of the elephant had a persistent germ, continuing its function through life, analogous to the growth of endogenous plants. In the first place, there could be no pus without infection. At the point of infection microbes had access to it, without which there could be no pus. When the tooth of the elephant was in quite an early stage of development, a wound in the bone near the base of the pulp might give ingress to microbes, bringing about pus infection; in the continuous growth of the tooth, subsequent layers of dentine might coalesce, forming a solid tusk in its elongation, the cavity and its encysted pus being carried forward with its growth until entirely covered in the pus cavity, being thus found at last in the solid portion of the tusk. In others a bridge of dentine may have been formed across, leaving the cavity open. New dentine-forming cells may be organized for the formation of the bridge across the cavity, or the cavity may be obliterated when there is no longer any pouring out of indifferent corpuscles, melting down under the influence of microbes.

Dr. Baldwin, of Chicago, said that it had been asserted positively that micro-organisms were necessary to the formation of pus, and no one had protested. Though it is true that there are micro-organisms everywhere, in the air, etc., yet he took direct issue with the statement that they must be present in the formation of pus. In a felon, pus is formed beneath the periosteum. If the microbes get there through the medium of the circulation (the only way possible), then where is the use of germicides ?

Dr. Barrett said he did not think it necessary to argue that point in the present state of biological science.

Dr. A. H. Thompson, of Topeka, Kan., wished to suggest that precedence be given to foreign papers.

Dr. J. Hale Moore, of Richmond, Va., said that much valuable time had been lost in talk that was not scientific, in wrangling as to priority of invention, etc. He thought the section should be guided strictly by the medical code of ethics. He wished to offer a resolution that all papers giving modes of operating, or cuts, diagrams or models of patents, and also their discussion, should be excluded. President Taft said that the editor of the transactions would decide those points.

Dr. Dudley (Salem, Mass.) said there was nothing in the ethics of the medical profession preventing a surgeon from patenting his appliances. He moved that the resolution be laid on the table.

A division being called for, the motion to table was lost and the resolution passed.

Adjourned.

